# Rapid improvement in spinal pain in patients with axial spondyloarthritis treated with secukinumab: primary results from a randomized controlled phase-IIIb trial

**DOI:** 10.1177/1759720X211051471

**Published:** 2021-10-22

**Authors:** Denis Poddubnyy, Effie Pournara, Agnieszka Zielińska, Asta Baranauskaite, Alejandro Muñoz Jiménez, Sanchayita Sadhu, Barbara Schulz, Michael Rissler, Chiara Perella, Helena Marzo-Ortega

**Affiliations:** Head of the Rheumatology Unit, Department of Gastroenterology, Infectious Diseases and Rheumatology (including Nutrition Medicine), Charité – Universitätsmedizin Berlin, Campus Benjamin Franklin, Hindenburgdamm 30, 12203 Berlin, Germany; Department of Epidemiology, German Rheumatism Research Centre, Berlin, Germany; Novartis AG, Basel, Switzerland; Medycyna Kliniczna, Warszawa, Poland; Lithuanian University of Health Sciences, Kaunas, Lithuania; Hospital Universitario Virgen del Rocío, Sevilla, Spain; Novartis Healthcare Private Limited, Hyderabad, India; Novartis AG, Basel, Switzerland; Novartis AG, Basel, Switzerland; Novartis AG, Basel, Switzerland; NIHR Leeds Biomedical Research Centre, LTHT and LIRMM, University of Leeds, Leeds, UK

**Keywords:** axial spondyloarthritis, interleukin-17A inhibitor, spinal pain, tumour necrosis factor inhibitor

## Abstract

**Background:**

This study aimed to evaluate the efficacy and safety of secukinumab 150 mg compared with placebo in the management of spinal pain and disease activity in patients with axial spondyloarthritis (axSpA) at Week 8 and up to Week 24.

**Methods:**

Patients (*n* = 380) with active axSpA were randomized (3:1) to secukinumab 150 mg (Group A) or placebo (Group B). At Week 8, patients from Group A with an average spinal pain score <4 were defined as responders and were re-assigned to secukinumab 150 mg (Arm A1); whereas non-responders were re-randomized to secukinumab 150/300 mg (Arm A2/A3). Patients from Group B were re-randomized (1:1) to secukinumab 150/300 mg (Arm B1/B2).

**Results:**

At Week 8, the odds of achieving an average spinal pain score of <4 were significantly higher for patients on secukinumab 150 mg than for patients on placebo (odds ratio (OR): 1.89; 95% confidence interval (CI): 1.08–3.33; *p* = 0.0264). Further reductions in spinal pain were observed across treatment groups up to Week 24. Pronounced improvements were also observed in other disease activity measurements, such as Bath Ankylosing Spondylitis Disease Activity Index and Ankylosing Spondylitis Disease Activity Score. Responders from Group A showed the highest improvements for all measured parameters of spinal pain compared with the other arms. No new or unexpected safety signals were observed.

**Conclusion:**

Secukinumab provided rapid and significant improvement in spinal pain at Week 8 which was sustained or increased further up to Week 24 in patients with axSpA.

**Trial Registration:**

ClinicalTrials.gov: NCT03136861. Registered May 2, 2017.

## Introduction

Axial spondyloarthritis (axSpA) is a chronic inflammatory condition that primarily affects the spine and the sacroiliac joints, with a reported global prevalence of around 1%.^1–3^ AxSpA can be classified into radiographic (r-axSpA, also termed ankylosing spondylitis (AS)) or non-radiographic (nr-axSpA) based on the presence of radiographic sacroiliitis as per the radiographic criterion of the modified New York criteria for AS.^2^

Chronic back pain is the cardinal symptom of axSpA and, although multifactorial, is related to inflammation in the sacroiliac joints and in the spine. Inflammation is followed by repair and new bone formation, resulting in spinal ankylosis that is associated with mobility restrictions in the axial skeleton. This contributes to increased disease burden and limits the performance of daily activities in individuals with axSpA.^2^ The European Map of Axial Spondyloarthritis study analysed patient perspectives of axSpA in 13 countries and reported that patients expressed fear of disease progression and suffering from pain in addition to the loss of functional mobility. Patients hoped to halt disease progression and mitigate pain with effective treatment options.^4^

Treatment strategies in axSpA aim to reduce inflammation, control signs, and symptoms, such as pain and stiffness, and prevent or delay the progression of structural damage in the spine that could result in preservation of functional status and improvements in quality of life (QoL) in the long term.^5^ Physical therapy and treatment with non-steroidal anti-inflammatory drugs (NSAIDs) are considered the first line of treatment in axSpA,^6^ followed by biological disease-modifying anti-rheumatic drugs (bDMARDs), such as tumour necrosis factor inhibitors (TNFis) and interleukin (IL)-17 inhibitors.^6–9^ Secukinumab, a fully human monoclonal antibody that directly inhibits IL-17A, has shown significant and sustained improvements in the signs and symptoms across the nr- and r-axSpA disease spectrum in several phase-III trials.^10–13^

Despite pain being the most troubling symptom for the patients, clinical studies in axSpA routinely use composite disease activity measures to assess the treatment effect.^6,9^ SKIPPAIN (NCT03136861) is the first randomized controlled study to evaluate spinal pain as the primary endpoint in patients with axSpA as early as Week 8.^14^

## Methods

### Patients

Male and female patients of ⩾18 years with a diagnosis of axSpA, classified as nr-axSpA or r-axSpA according to the Assessment of SpondyloArthritis international Society (ASAS) criteria,^9^ active spinal disease defined by the Bath Ankylosing Spondylitis Disease Activity Index (BASDAI) score of ⩾4 and average spinal pain score of >4 were enrolled in the trial. Patients should have had an inadequate response to the highest recommended dose of at least two NSAIDs over a period of ⩾4 weeks; in the case of toxicity, intolerance, or contraindications, this period was shorter. Patients on regular NSAIDs as part of their axSpA therapy were required to be on a stable dose for ⩾2 weeks before randomization. Patients previously on a TNFi were also allowed to enter the trial but should have had an appropriate washout period prior to randomization.

Key exclusion criteria included previous treatment or exposure to any biological immunomodulating agents, except one TNF-α inhibitor agent; current and active inflammatory diseases other than axSpA; any mechanical disease that affects the spine; any active systemic infections; history of chronic or recurrent infectious disease; history of any known malignancy within the past 5 years; serious medical conditions, such as uncontrolled hypertension or congestive heart failure; and any underlying conditions that could compromise the immune system. Detailed inclusion and exclusion criteria are listed in Supplementary Table S1.

### Patient and public involvement

Representatives from Patient Associations were members of the scientific steering committee that designed the study. The study protocol was reviewed by the Independent Ethics Committee or Institutional Review Board for each participating centre. The study was conducted according to the International Council for Harmonization of Technical Requirements for Pharmaceuticals for Human Use (ICH) E6 Guideline for Good Clinical Practice that has its origin in the Declaration of Helsinki.^15^ Written informed consent was obtained from each enrolled participant.

### Study design

SKIPPAIN is a randomized, double-blind, placebo-controlled, and multicenter study. The study consisted of two treatment periods: a double-blind, placebo-controlled period from baseline to Week 8 (Treatment Period 1 (TP1) and a double-blind secukinumab treatment period from Week 8 to Week 24 (Treatment Period 2 (TP2)) This study was initiated on June 30, 2017 (first patient and first visit) and completed on February 15, 2019 (last patient and last visit), and was conducted across 66 sites in 17 countries.

### Randomization and blinding

Eligible patients were randomized (3:1) to receive either secukinumab 150 mg (Group A) or placebo (Group B) in TP1 at baseline and at Weeks 1, 2, 3, and 4 (Supplementary Figure 1). At Week 8, patients entered TP2 and were re-randomized or re-assigned to one of the five treatment arms (Arms A1, A2, A3, B1, and B2). Responders from Group A, defined as achieving an average spinal pain score <4, continued with secukinumab 150 mg and were re-assigned to Arm A1, whereas non-responders were re-randomized either to secukinumab 150 mg (Arm A2) or to secukinumab 300 mg (Arm A3) using an up-titration approach. Patients from Group B were re-randomized to either secukinumab 150 mg (Arm B1) or secukinumab 300 mg (Arm B2) in a 1:1 ratio.

Study treatment was administered by subcutaneous injections using 1 mL pre-filled syringes throughout the study. The identity of the treatments was concealed using study drugs (active and placebo) that were all identical in packaging, labelling, schedule of administration, and appearance.

### Outcome measures

The primary objective of this study was to assess the superiority of secukinumab 150 mg to placebo in achieving an average spinal pain score of <4 on a 0–10 numerical rating scale (NRS) at Week 8. The spinal pain NRS is an 11-point scale to assess the pain intensity in patients who are able to self-report. The average spinal pain score is the average of the total score (pain at any time) and the nocturnal score (pain during the night). The patient was asked to answer two questions in order to make two pain ratings corresponding to the intensity of spinal pain at any time and the intensity of the pain during the night. The secondary objective was to assess the superiority of secukinumab 150 mg to placebo in achieving a BASDAI score of <4 at Week 8.

The exploratory objectives were the proportion of patients achieving a spinal pain score of <4 (NRS) in Group A compared with Group B at Weeks 1, 2, 3, and 4, and in each treatment arm at Week 24; BASDAI score of <4 in each treatment arm at Week 24; and Ankylosing Spondylitis Disease Activity Score (ASDAS) <2.1 and ASDAS <1.3 in Group A compared with Group B at Week 8, and in each treatment arm at Week 24; in addition to mean change from baseline in the Functional Assessment of Chronic Illness Therapy-Fatigue (FACIT-Fatigue) score in Group A compared with Group B at Week 8, and in each treatment arm at Week 24; and ASAS health index (ASAS-HI) in each treatment arm at Week 24.

The overall safety and tolerability of secukinumab 150 and 300 mg were assessed by adverse events (AEs), serious adverse events (SAEs), and AEs of special interest in both TP1 and TP2.

### Statistical analysis

Based on the average response rates (spinal pain (NRS) < 4) from the MEASURE 2^16^ and MEASURE 3^13^ trials at Week 16, 43.6% and 23.5% of the patients were assumed as responders for the secukinumab and the placebo groups, respectively. To achieve 90% power on a two-sided 5% significance level, with 3:1 (secukinumab: placebo) allocation and accounting for patients dropping out or those with protocol deviations, 352 patients (264 in the secukinumab group and 88 in the placebo group) were required.

The full analysis set (FAS) of TP1 (FAS-TP1) consisted of all patients who were randomized into this study at baseline and received at least one dose of study treatment during this treatment period. The FAS of TP2 (FAS-TP2) consisted of all patients who were re-randomized/re-assigned at Week 8 and received at least one dose of treatment during this treatment period.

The primary analysis, based on the FAS-TP1 patients, was conducted via a logistic regression model with treatment, country and the stratification factor of prior exposure to TNFi as covariates. The odds ratio (OR), its 95% confidence interval (CI), and *p* values were calculated by comparing the secukinumab 150 mg treatment group with the placebo group at Week 8. The pain scores were analysed using a repeated measures analysis of variance (ANOVA) model. The model included the same factors as the logistic regression model of the primary analysis plus the baseline score as a covariate. Analysis visit was used as a repeat factor.

The secondary analysis was based on FAS-TP1 patients and was performed similarly to the primary analysis. The evaluation of exploratory efficacy variables was performed on the FAS-TP1 and FAS-TP2 populations based on the corresponding treatment periods. As the exploratory analyses were outside of the confirmatory framework, formal statistical tests were not performed and therefore, *p* values were not calculated and conclusions of statistical inference were not drawn.

Safety analyses were performed on the safety set and were presented separately for TP1 and TP2. The safety set included all patients who received at least one dose of study treatment during the given treatment period. The safety follow-up was carried forward until 12 weeks after the last study treatment.

## Results

Of 448 patients who were screened for eligibility, 65 (14.5%) patients discontinued during screening either due to not meeting the eligibility criteria of selection (10.9%), patient’s/guardian’s decision (1.1%), occurrence of AEs (0.4%), loss to follow-up (0.4%), or withdrawal of informed consent (1.6%). A total of 380 patients were randomized with 285 patients in the secukinumab 150-mg group and 95 patients in the placebo group.

In total, 97.5% of patients in the secukinumab 150-mg group and 93.7% in the placebo group completed TP1. At the end of TP2, the completion rates ranged between 97.7% and 100% among the secukinumab 300-mg (Arm B2) and 150-mg (Arm B1) groups. Details on patient disposition up to Week 24 are presented in Supplementary Figure 2.

Demographics and baseline disease characteristics of the secukinumab 150-mg and placebo groups were comparable (Table 1). A total of 11.9% of patients in the secukinumab 150-mg group and 11.6% in the placebo groups were previously exposed to TNFi. The proportion of r-axSpA patients, based on radiographic evidence of sacroiliitis according to modified New York criteria as per the investigator’s judgement, was 70.5% and 71.6% in the secukinumab 150-mg and the placebo groups, respectively.

**Table 1.** Demographics and baseline disease characteristics.

Characteristic	Secukinumab 150 mg (*N* = 285)	Placebo (*N* = 95)	Total (*N* = 380)
Age (years), mean (SD)	42.3 (11.9)	40.9 (12.2)	42.0 (12.0)
Female, *n* (%)	106 (37.2)	39 (41.1)	145 (38.2)
Race (Caucasian), *n* (%)	267 (93.7)	93 (97.9)	360 (94.7)
BMI (kg/m^2^), mean (SD)	27.0 (5.2)	27.2 (5.8)	27.0 (5.3)
r-axSpA, *n* (%)^a^	201 (70.5)	68 (71.6)	269 (70.8)
Uveitis, *n* (%)	46 (16.1)	12 (12.6)	58 (15.3)
IBD, *n* (%)	2 (0.7)	0	2 (0.5)
Psoriasis, *n* (%)	22 (7.7)	7 (7.4)	29 (7.6)
Dactylitis, *n* (%)	21 (7.4)	6 (6.3)	27 (7.1)
Enthesitis, *n* (%)	72 (25.3)	28 (29.5)	100 (26.3)
Peripheral arthritis, *n* (%)	104 (36.5)	30 (31.6)	134 (35.3)
Family history of spondyloarthritis, *n* (%)	45 (15.8)	16 (16.8)	61 (16.1)
HLA-B27 positive, *n* (%)	233 (81.8)	76 (80.0)	309 (81.3)
Abnormal C-reactive protein (>5 mg/L), *n* (%)	140 (49.1)	49 (51.6)	189 (49.7)
Time since diagnosis of axSpA (years), mean (SD)	6.7 (8.6)	5.5 (7.2)	6.4 (8.3)
Time since onset of back pain (years), mean (SD)	13.2 (10.1)	12.3 (9.6)	12.9 (10.0)
Spinal pain NRS score (average), mean (SD)	7.3 (1.4)	7.3 (1.3)	7.3 (1.4)
Spinal pain NRS score (total), mean (SD)	7.3 (1.4)	7.4 (1.3)	-
Spinal pain NRS score (nocturnal), mean (SD)	7.2 (1.5)	7.2 (1.5)	-
BASDAI score, mean (SD)	7.1 (1.2)	6.9 (1.4)	7.0 (1.3)
ASDAS, mean (SD)	3.7 (0.9)	3.7 (0.8)	3.7 (0.9)
PGA of disease activity, mean (SD)	7.5 (1.4)	7.2 (1.7)	7.4 (1.5)
FACIT-Fatigue, mean (SD)	21.6 (9.0)	22.6 (9.0)	21.8 (9.0)
ASAS health index, mean (SD)	10.4 (3.6)	10.1 (3.6)	10.3 (3.6)
Previous exposure to TNF inhibitors, *n* (%)	34 (11.9)	11 (11.6)	45 (11.8)

AS, ankylosing spondylitis; ASAS, Assessment of SpondyloArthritis international Society; ASDAS, Ankylosing Spondylitis Disease Activity Score; axSpA, axial spondyloarthritis; BASDAI, Bath Ankylosing Spondylitis Disease Activity Index; BMI, body mass index; FACIT-Fatigue, Functional Assessment of Chronic Illness Therapy-Fatigue; HLA, human leukocyte antigen; IBD, inflammatory bowel disease; mNY criteria, modified New York criteria; N, total number of randomized patients; n, number of patients with observations; NRS, numerical rating scale; nr-axSpA, non-radiographic axial spondyloarthritis; PGA, Patient Global Assessment; r-axSpA, radiographic axial spondyloarthritis; SD, standard deviation; TNF, tumour necrosis factor.

a Based on radiographic evidence for sacroiliitis according to the mNY criteria as per the investigator’s judgement.

### Efficacy

#### Treatment Period 1

At Week 8, the responder rates were 31.9% for those treated with secukinumab 150 mg and 20.0% for those treated with placebo. The odds of being a responder on the average spinal pain score were, therefore, significantly higher for a patient on secukinumab 150 mg than on placebo (OR: 1.89; 95% CI: 1.08–3.33; *p* = 0.0264).

**Figure 1.**
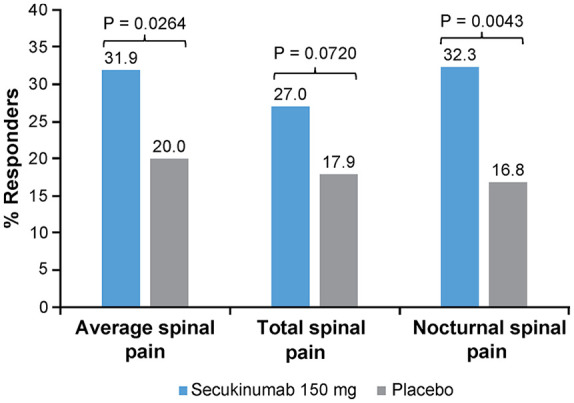
Proportion of patients with spinal pain (average, nocturnal, and total) NRS score <4 (responders) at Week 8.

The odds of being a responder on the nocturnal spinal pain score in the secukinumab 150-mg group were significantly higher (OR: 2.38; 95% CI: 1.31–4.31; *p* = 0.0043) compared with the placebo group, whereas the odds were higher for the total spinal pain score although not statistically significant (OR: 1.72; 95% CI: 0.95–3.10; *p* = 0.0720; Figure 1). The proportion of responders was higher in the secukinumab 150-mg group compared with placebo as early as Week 1 (9.8% *vs* 2.1%) and up to Week 8 (31.9% *vs* 20.0%).

**Table:** 

Variable	Odds ratio versus placebo	95% CI
Spinal pain NRS score (average)	1.89	(1.08, 3.33)
Spinal pain NRS score (total)	1.72	(0.95, 3.10)
Spinal pain NRS score (nocturnal)	2.38	(1.31, 4.31)

CI, confidence interval; NRS, numerical rating scale.

At Week 8, the proportion of patients with a BASDAI score of <4 was 33.3% in the secukinumab 150-mg group and 23.2% in the placebo group. The odds of achieving a BASDAI score of <4 at Week 8 based on the responder rates were therefore significantly higher for patients on secukinumab 150 mg than for patients on placebo (OR: 1.75; 95% CI: 1.01–3.04; *p* = 0.0466). From baseline to Week 8, the mean BASDAI scores showed a higher gradual improvement in the secukinumab 150-mg group *versus* placebo (7.09–4.82 *vs* 6.92–5.51). A higher improvement (decrease) in mean ASDAS was also observed from baseline to Week 8 in the secukinumab 150-mg group *versus* placebo (3.75–2.52 *vs* 3.67–3.19).

#### Treatment Period 2

**Figure 2.**
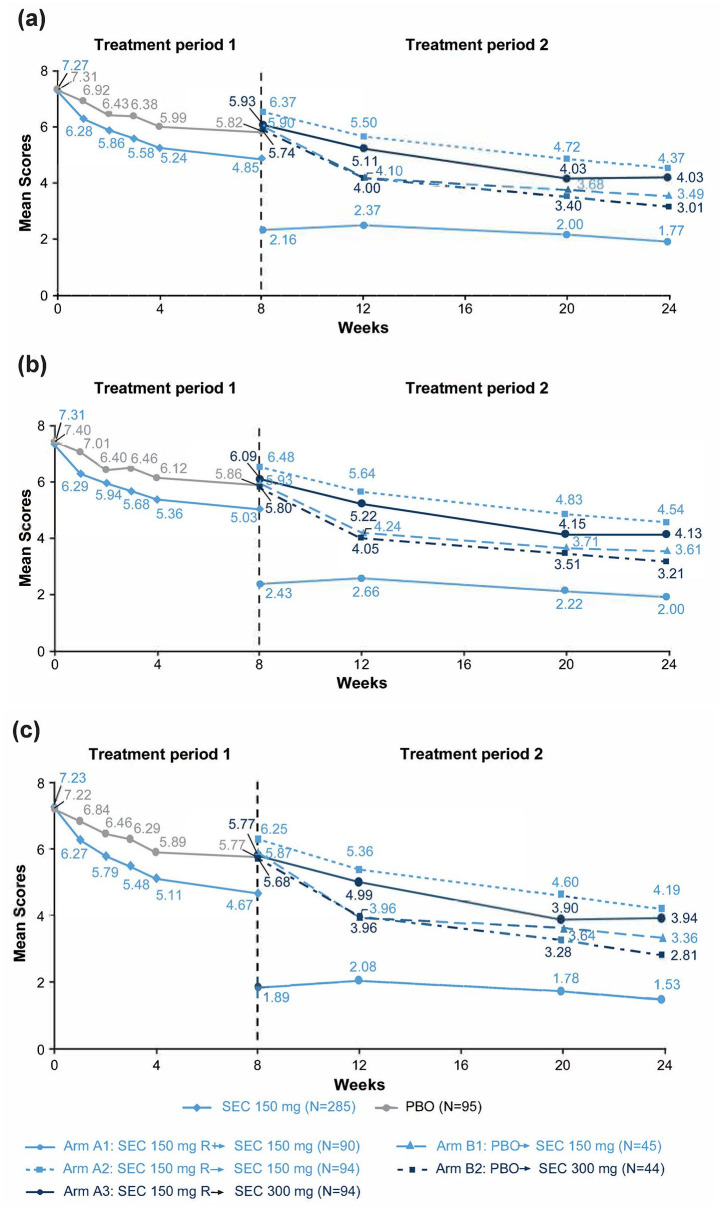
Spinal pain score—(A) average, (B) total, and (C) nocturnal in patients with axial spondyloarthritis in Treatment Period 1 and Treatment Period 2.

Although improvements in pain scores were observed in all treatment arms at the end of TP2, responders who continued on secukinumab 150 mg (Arm A1) showed the highest improvements (reduction) for the mean average pain score (1.77), total pain score (2.00), and nocturnal pain score (1.53) compared with the other groups (Figure 2).

**Table:** 

TP 1 (up to Week 8)	TP 2 (Week 8–Week 24)
SEC 150 mg	Arm A1: SEC 150 mg responders (average spinal pain score < 4) at Week 8 re-assigned to continue SEC 150 mg every 4 weeks
	Arm A2: SEC 150 mg non-responders (average spinal pain score ⩾ 4) re-randomized to continue SEC 150 mg every 4 weeks
	Arm A3: SEC 150 mg non-responders (average spinal pain score ⩾ 4) were up-titrated to SEC 300 mg every 4 weeks
PBO	Arm B1: Patients randomized to PBO at baseline were re-randomized to SEC 150 mg every 4 weeks
	Arm B2: Patients randomized to PBO at baseline were re-randomized to SEC 300 mg every 4 weeks

*N*, total number of randomized patients; PBO, placebo; R+, responders; R−, non-responders; SEC, secukinumab; TP, treatment period.

Responders who continued on secukinumab 150 mg (Arm A1) also showed the highest improvements in BASDAI score and ASDAS. BASDAI mean reductions from Week 8 in non-responders who continued on secukinumab 150 mg (Arm A2) and 300 mg (Arm A3) were −1.53 and −1.62, and the ASDAS mean reductions from baseline were −1.24 and −1.47, respectively. In placebo patients who received secukinumab 150 mg (Arm B1) and 300 mg (Arm B2), BASDAI mean reductions from Week 8 were −2.06 and −2.23, and ASDAS mean reductions from baseline were −1.49 and −1.77, respectively.

#### ASDAS < 2.1/ASDAS < 1.3, FACIT-Fatigue and ASAS-HI for TP1 and TP2

**Figure 3.**
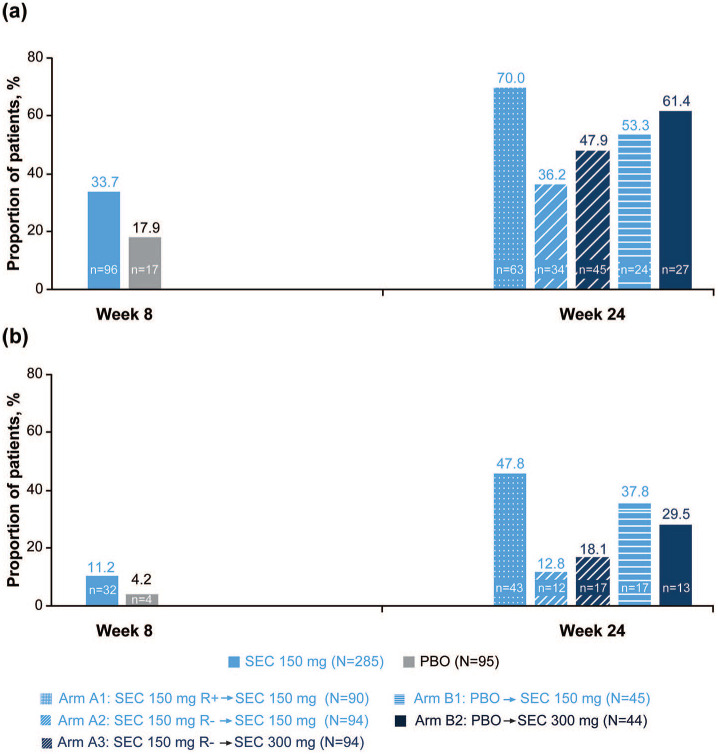
Proportion of patients achieving (A) low disease activity (ASDAS < 2.1) and (B) inactive disease (ASDAS < 1.3) according to ASDAS-CRP in Treatment Period 1 (Week 8) and Treatment Period 2 (Week 24).

ASDAS < 2.1 (low disease activity) and ASDAS < 1.3 (inactive disease) responses in TP1 and TP2 are presented in Figure 3. The proportion of patients with an ASDAS of <1.3 was higher in the secukinumab 150-mg group compared with the placebo group (11.2% *vs* 4.2%) in TP1; in TP2, the proportion was highest in patients who continued on secukinumab 150 mg (Arm A1; 47.8%) than in the other treatment arms.

**Table:** 

TP 1 (up to Week 8)	TP 2 (Week 8–Week 24)
SEC 150 mg	Arm A1: SEC 150 mg responders (average spinal pain score < 4) at Week 8 re-assigned to continue SEC 150 mg every 4 weeks
	Arm A2: SEC 150 mg non-responders (average spinal pain score ⩾ 4) re-randomized to continue SEC 150 mg every 4 weeks
	Arm A3: SEC 150 mg non-responders (average spinal pain score ⩾ 4) were up-titrated to SEC 300 mg every 4 weeks
PBO	Arm B1: Patients randomized to PBO at baseline were re-randomized to SEC 150 mg every 4 weeks
	Arm B2: Patients randomized to PBO at baseline were re-randomized to SEC 300 mg every 4 weeks

ASDAS, Ankylosing Spondylitis Disease Activity Score; *N*, total number of randomized patients; n, number of patients with (A) ASDAS < 2.1 and (B) ASDAS < 1.3; PBO, placebo; R+, responders; R−, non-responders; SEC, secukinumab; TP, treatment period.

**Figure 4.**
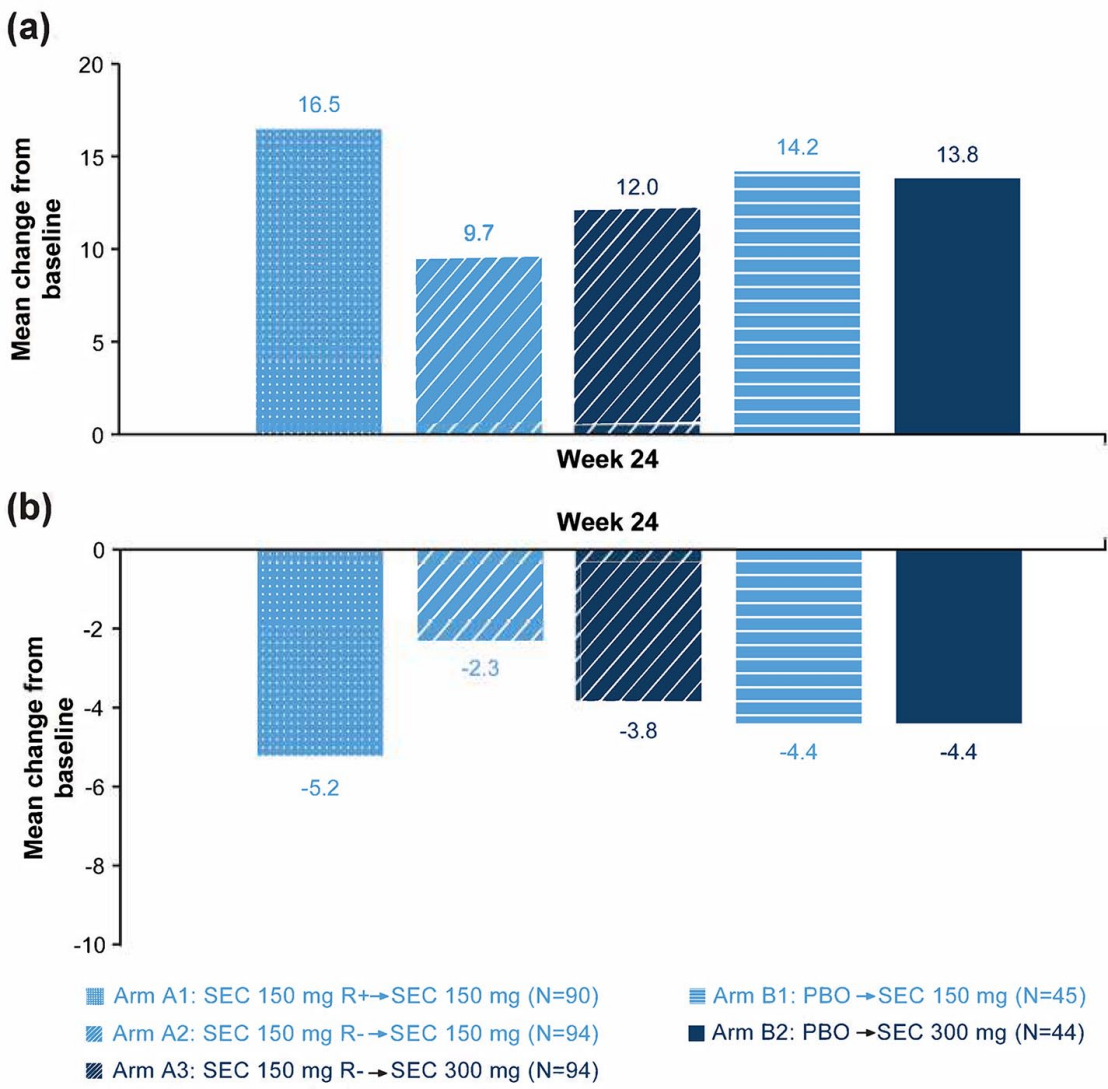
Mean change from baseline scores of (A) FACIT-Fatigue and (B) ASAS-HI with secukinumab treatment in the entire treatment period.

An improvement in the FACIT-Fatigue score was observed in all treatment arms compared to baseline in TP2, and the mean increase was highest in patients who continued on secukinumab 150 mg (Arm A1; 16.48). In all treatment arms, improvement (decrease) in ASAS-HI from baseline was observed at Week 24, and Arm A1 showed the highest mean improvement (−5.2) (Figure 4).

**Table:** 

TP 2 (Week 8–Week 24)
Arm A1: SEC 150 mg responders (average spinal pain score < 4) at Week 8 re-assigned to continue SEC 150 mg every 4 weeks
Arm A2: SEC 150 mg non-responders (average spinal pain score ⩾ 4) re-randomized to continue SEC 150 mg every 4 weeks
Arm A3: SEC 150 mg non-responders (average spinal pain score ⩾ 4) were up-titrated to SEC 300 mg every 4 weeks
Arm B1: Patients randomized to PBO at baseline were re-randomized to SEC 150 mg every 4 weeks
Arm B2: Patients randomized to PBO at baseline were re-randomized to SEC 300 mg every 4 weeks

ASAS, Assessment of SpondyloArthritis international Society; FACIT-Fatigue, Functional Assessment of Chronic Illness Therapy-Fatigue; HI, health index; N, total number of randomized patients; PBO, placebo; R +, responders; R-, non-responders; SEC, secukinumab.

The proportion of patients with r-axSpA *versus* nr-axSpA that reported an average spinal pain (NRS < 4) was 30.3 *versus* 35.7 in the secukinumab 150-mg group at Week 8, and the proportion was highest for both categories in Arm A1 (83.3 *vs* 86.7) at Week 24; and those reported a BASDAI score < 4 at Week 8 was 35.3 *versus* 28.6 and at Week 24 was 86.7 *versus* 76.7 (Arm A1).

### Safety

A summary of safety results from TP1 and TP2 is presented in Table 2. In TP1, the mean (standard deviation (SD) duration of exposure was 28.9 (2.96) days and was comparable between secukinumab 150-mg and placebo groups (29.0 (2.48) days *vs* 28.7 (4.09) days). During TP2, the majority of patients (83.9%) were exposed to study treatment for ⩾12 weeks. The mean duration of exposure during this period for the overall population was 84.2 (8.35) days and was comparable across the five treatment arms.

**Table 2.** Safety profile up to Week 8 and for the entire treatment period.

	Treatment Period 1		Treatment Period 2					Total (*N* = 380)
	Secukinumab 150 mg (*N* = 285)	Placebo (*N* = 95)	Arm A1 (*N* = 90)	Arm A2 (*N* = 94)	Arm A3 (*N* = 94)	Arm B1 (*N* = 45)	Arm B2 (*N* = 44)	
Duration of exposure (days), mean (SD)	29.0 (2.48)	28.7 (4.09)	84.5 (7.33)	83.6 (9.34)	84.2 (9.54)	85.5 (2.40)	83.4 (9.34)	84.2 (8.35)^a^
Any AE, *n* (%)	99 (34.74)	25 (26.32)	34 (37.78)	34 (36.17)	30 (31.91)	9 (20.00)	13 (29.55)	183 (48.16)
Serious AEs, *n* (%)	4 (1.40)	0	3 (3.33)	1 (1.06)	1 (1.06)	0	0	9 (2.37)
AEs of special interest, *n* (%)	41 (14.39)	13 (13.68)	15 (16.67)	12 (12.77)	15 (15.96)	3 (6.67)	5 (11.36)	88 (23.16)
Discontinued due to AEs, *n* (%)	3 (1.05)	2 (2.11)	0	1 (1.06)	2 (2.13)	0	1 (2.27)	9 (2.37)
Death, *n* (%)	0	0	0	0	0	0	0	0
Overall most common AEs	n (%)		EAIR (95% CI)					
Nasopharyngitis	6 (2.11)	1 (1.05)	2.91 (0.07, 16.20)	8.38 (1.73, 24.49)	11.15 (3.04, 28.54)	11.50 (1.39, 41.54)	11.92 (1.44, 43.06)	6.40 (3.79, 10.12)
Headache	10 (3.51)	1 (1.05)	5.80 (0.70, 20.93)	5.57 (0.67, 20.12)	8.35 (1.72, 24.41)	0.00 (0.00, 21.18)	5.92 (0.15, 32.99)	6.43 (3.81, 10.17)
Oropharyngeal pain	8 (2.81)	1 (1.05)	11.63 (3.17, 29.79)	5.56 (0.67, 20.10)	0.00 (0.00, 10.21)	0.00 (0.00, 21.18)	0.00 (0.00, 21.83)	4.63 (2.46, 7.91)
Upper respiratory tract infection	4 (1.40)	5 (5.26)	2.89 (0.07, 16.09)	0.00 (0.00, 10.22)	8.36 (1.72, 24.44)	0.00 (0.00, 21.18)	0.00 (0.00, 21.83)	4.27 (2.21, 7.46)
Arthralgia	4 (1.40)	2 (2.11)	0.00 (0.00, 10.66)	8.43 (1.74, 24.64)	2.78 (0.07, 15.47)	0.00 (0.00, 21.18)	0.00 (0.00, 21.83)	3.56 ( 1.71, 6.54)
Blood creatinine increased	4 (1.40)	0	8.77 (1.81, 25.62)	2.76 (0.07, 15.41)	0.00 (0.00, 10.21)	0.00 (0.00, 21.18)	12.15 (1.47, 43.89)	3.54 (1.70, 6.52)
Diarrhoea	6 (2.11)	1 (1.05)	0.00 (0.00, 10.66)	0.00 (0.00, 10.22)	2.77 (0.07, 15.43)	0.00 (0.00, 21.18)	0.00 (0.00, 21.83)	2.84 (1.23, 5.60)
AEs of special interest	n (%)		EAIR (95% CI)					
Infections	33 (11.58)	11 (11.58)	38.74 (20.63, 66.25)	31.53 (15.74, 56.41)	40.25 (22.01, 67.53)	17.44 (3.60, 50.97)	30.67 (9.96, 71.57)	29.19 (23.08, 36.44)
Ulcerative colitis	1 (0.35)	0	5.82 (0.71, 21.04)	2.78 (0.07, 15.49)	0.00 (0.00, 10.21)	0.00 (0.00, 21.18)	0.00 (0.00, 21.83)	1.41 (0.38, 3.61)
Crohn’s disease	0	0	-	-	-	-	-	-
MACE^b^	2 (0.70)	0	0.00 (0.00, 10.66)	2.79 (0.07, 15.52)	0.00 (0.00, 10.21)	0.00 (0.00, 21.18)	0.00 (0.00, 21.83)	1.06 (0.22, 3.09)
Malignancy	0	0	0.00 (0.00, 10.66)	0.00 (0.00, 10.22)	2.77 (0.07, 15.42)	0.00 (0.00, 21.18)	0.00 (0.00, 21.83)	0.35 (0.01, 1.96)
Neutro analysis of variance penia	1 (0.35)	1 (1.05)	2.91 (0.07, 16.21)	0.00 (0.00, 10.22)	0.00 (0.00, 10.21)	0.00 (0.00, 21.18)	5.92 (0.15, 32.97)	1.41 (0.39, 3.62)
Uveitis	0	0	2.89 (0.07, 16.08)	2.78 (0.07, 15.50)	0.00 (0.00, 10.21)	0.00 (0.00, 21.18)	0.00 (0.00, 21.83)	0.70 (0.09, 2.55)

AE, adverse events; CI, confidence interval; EAIR, exposure-adjusted incidence rates/100 subject years; MACE, major adverse cardiovascular events; *N*, number of randomized patients; *n*, number of patients with AEs; NRS, numerical rating scale; SD, standard deviation.

At Week 8, patients entered Treatment Period 2 and received either secukinumab 150 or 300 mg every 4 weeks starting at Week 8 up to Week 20.

Arm A1: Patients on secukinumab 150 mg who achieved spinal pain NRS score < 4 at Week 8 re-assigned to continue secukinumab 150 mg every 4 weeks; Arm A2: Patients on secukinumab 150 mg who did not achieve spinal pain NRS score < 4 at Week 8 re-randomized to continue secukinumab 150 mg every 4 weeks; Arm A3: Patients on secukinumab 150 mg who did not achieve spinal pain NRS score <4 at Week 8 re-randomized to escalate to secukinumab 300 mg every 4 weeks; Arm B1: Patients randomized to placebo at baseline re-randomized to secukinumab 150 mg every 4 weeks; Arm B2: Patients randomized to placebo at baseline re-randomized to secukinumab 300 mg every 4 weeks.

a *N* = 367 (total of Treatment Period 2 only).

b MACE includes myocardial infarction and acute myocardial infarction.

Overall, nine patients (2.4%) who received secukinumab and/or placebo experienced one or more SAEs over the entire treatment period, of them; four (1.4%) of these patients reported SAEs in the secukinumab 150-mg group in TP1. In the entire treatment period, nine (2.4%) patients discontinued due to AEs, three (1.1%) of whom in the secukinumab 150-mg group during TP1.

Headache was the most common AE reported across all treatment arms, and the exposure-adjusted incidence rate (EAIR)/100 subject-years (95% CI) was 6.43 (3.81–10.17), followed by nasopharyngitis (6.40 (3.79–10.12)), oropharyngeal pain (4.63 (2.46–7.91)), and upper respiratory tract infection (4.27 (2.21–7.46)). Of the AEs of special interest, the EAIRs for ulcerative colitis and major adverse cardiovascular events were 1.41 (0.38–3.61) and 1.06 (0.22–3.09), respectively. There were four (1.05%) cases of neutropenia and two (0.53%) cases of uveitis reported during the entire treatment period. No deaths were reported in the entire treatment period of 24 weeks.

## Discussion

SKIPPAIN showed that secukinumab 150 mg was effective in reducing spinal pain and improving disease activity measures in patients with axSpA as early as Week 8. So far, pain has been assessed as an exploratory endpoint in axSpA trials and has never been studied as the primary endpoint. Therefore, clinical trials mirroring daily clinical practice to the best extent possible and applying a clinically relevant testing strategy by prioritizing patient-relevant outcomes such as pain, are sparse. To answer this research question, pain was chosen over other ‘conventional’ endpoints, such as ASAS20/40 or partial remission in SKIPPAIN, in order to focus on patients’ priorities for treatment goals, and hence, it is the first randomized controlled study involving a bDMARD, with spinal pain as the primary endpoint in a broad population of patients across the axSpA spectrum (both r-axSpA and nr-axSpA).

As several studies have documented the impact of pain on the life of patients, both on physical and mental well-being, the early management of spinal pain and disease activity in patients with axSpA who have an inadequate response to prior NSAIDs was the main focus of SKIPPAIN.^17–19^ Patients with axSpA report impaired physical activity mainly due to pain and stiffness, fatigue, and poor sleep, and the burden of disease in axSpA is mainly driven by pain due to its multiple consequences on patients’ physical functioning and QoL.^20,21^ Furthermore, chronic pain, as a feature of axSpA, has been found to be associated with depression.^22^ Although the average spinal pain which is the average of the total score (pain at any time) and the nocturnal score (pain during the night) was used as the primary endpoint, separate assessments of the total and the nocturnal pain were collected at all timepoints in this study. It is worth noting that the significant improvements in nocturnal pain are of particular relevance as awakening because of back pain during the second half of the night and disturbed sleep is well recognized as the most troublesome symptom of patients with axSpA.

In an early reference to the problems of sleep in AS, the secretary for the National Ankylosing Spondylitis Society wrote, ‘it is at night that the spondylitic feels his condition most and is most conscious of the skeletal prison within him’.^23^ This contributes to daytime fatigue, which is likely to impact the ability and willingness of patients to engage in physical activity.^24^ Decreased night pain was also the most significant predictor of improvement in observed Jenkins Sleep Evaluation Questionnaire (JSEQ) scores and improvements in sleep quality significantly correlated with improvements in Short Form-36 (SF-36), BASFI, night back pain, BASDAI, and total back pain scores, as well as in measures of fatigue from the BASDAI and vitality from the SF-36.^25,26^

This study assessed patient-reported pain levels (spinal pain score < 4 on a NRS) and disease activity (BASDAI score < 4) as potential decision factors for treatment optimization. The cut-off value of spinal pain of <4 on an NRS was chosen based on the existing literature on the cut-off points for mild, moderate, and severe pain on the NRS in patients with chronic musculoskeletal pain. Studies have reported that NRS score of ⩽5 corresponds to ‘mild’ pain in patients with chronic musculoskeletal pain in terms of pain-related interference with functioning; in patients with a low catastrophizing tendency, an NRS score of ⩽3 corresponds to ‘mild’ pain.^27^ However, there is a lack of consensus on the thresholds of spinal pain scores for different levels of pain severity. It is worth noting that the magnitude of improvement with secukinumab 150 mg *versus* placebo was more pronounced in terms of objective measurements of disease activity such as ASDAS < 2.1 than in the achievement of spinal pain score <4.

Dose escalation of secukinumab (from 75 to 150 mg) improved outcomes in patients with r-axSpA;^11^ however, there is limited evidence for dose escalation from 150 to 300 mg. The up-titration approach at Week 8 to assess the potential benefits of a dose increase to secukinumab 300 mg for non-responders, that is those patients who did not reach an average spinal pain score <4 at Week 8 (Arm A3) is another unique feature of SKIPPAIN. A similar approach was followed in patients assigned to the placebo group (Group B) in TP1 and re-assigned to secukinumab 300 mg in TP2 (Arm B2) to investigate a higher starting dose of secukinumab. The selection of Week 8 as the timepoint of assessment of the primary endpoint was based on the rationale to avoid unnecessary exposure to placebo for an extended period of time as secukinumab has shown in previous studies a pain-relieving effect as early as Week 8 or even earlier. Furthermore, the optimal timepoint of dose escalation in patients with axSpA is not defined and in general, the so-called ‘strategy’ trials are lacking.

The up-titration approach was assessed at Week 24; secukinumab 300 mg (Arm A3) provided moderately higher numerical benefit on spinal pain compared to secukinumab 150 mg (Arm A2) (average score: 4.37 *vs* 4.03) and disease activity index (BASDAI score: 4.65 *vs* 4.01). In terms of high-hurdle endpoints, such as ASDAS < 2.1 or <1.3, a clear numerical benefit was observed for Arm A3 when compared to Arm A2. The serum blood concentration of secukinumab might have contributed to the modest incremental improvements in the group of patients who were up-titrated. However, it is worth noting that the timepoint of up-titration might be too early to observe a treatment effect, as a full response to the dose of 150 mg was most probably not reached. This also explains the difference in the anticipated and the actual response rates in the study.

Furthermore, the loading regimen was not applied for the up-titration to 300 mg (Arm A3) potentially contributing to modest incremental improvements at Week 24. No loading after up-titration might also had resulted to a rather small difference in the serum drug concentration between 150 and 300 mg as compared to what we would have observed after full loading. Nevertheless, the results at Week 24 are purely exploratory, and as the study was not powered to compare the two doses of secukinumab (150 *vs* 300 mg), the better effect observed with the 300 mg dose is useful only within a hypothesis generating framework suggesting that the step-up approach might be beneficial for patients with a sub-optimal response to secukinumab 150 mg.

In patients with AS, disease activity scores such as BASDAI and ASDAS-CRP are significantly associated with back pain.^28,29^ Although pain matters the most from a patient perspective, it is subjective, can be caused by other comorbid conditions and is not fully understood. Potential reasons for residual pain include degenerative disease, mechanical pain, chronic pain syndrome, and/or pain sensitization.^17^ Furthermore, in the course of the disease, patients with axSpA, are at an increased risk of clinical vertebral fractures potentially leading to residual pain.^30^ In addition, there is a higher prevalence of fibromyalgia in patients with axSpA^31^ which may confound response to treatment. Finally, a misdiagnosis of fibromyalgia cannot be excluded.

At the end of the study treatment, improved FACIT-Fatigue scores and ASAS-HI observed in all treatment arms underlie the improvement in functioning and health in patients with axSpA. Clinical improvements in patients with r-axSpA shown with secukinumab treatment including improved FACIT-Fatigue scores up to 2 years in the MEASURE 2 study^16^ and up to 5 years in the MEASURE 1 extension study^11^ had a positive impact on daily activities and functioning in these patients. ASAS-HI is a composite measure used to quantify health in patients with AS,^32^ and an improvement of this index in patients treated with secukinumab could be related to their improved overall health status.

The limitations of the study include the lack of consensus on the threshold of 4 to define the primary endpoint of the average spinal pain score responders. Although clinically relevant medical and psychiatric conditions with potential impact on the study outcomes were excluded upfront, depressive signs, psychological distress and other factor potentially affecting disease perception were not specifically evaluated in the study. In addition, Week 8 as the timepoint for up-titration may not be optimal because improvements in outcomes were observed up to Week 24 in patients who continued with 150-mg dose of secukinumab. Furthermore, the trial was not powered to show significant improvements of the up-titration approach and hence the results do not provide compelling evidence in this respect.

In conclusion, the results of the SKIPPAIN trial demonstrated that secukinumab provided a rapid and significant improvement in spinal pain and reduced disease activity in patients with axSpA. The up-titration strategy might be beneficial for sub-optimal responders to secukinumab 150 mg. Reduction of spinal pain in patients with axSpA and early identification of non-responders may result in treatment optimization strategies and improved patient-related outcomes. “Residual” pain might still be an issue and a reason for some patients not achieving the treatment goal, warranting further investigation.

## Supplemental Material

sj-doc-1-tab-10.1177_1759720X211051471 – Supplemental material for Rapid improvement in spinal pain in patients with axial spondyloarthritis treated with secukinumab: primary results from a randomized controlled phase-IIIb trial Click here for additional data file. Supplemental material, sj-doc-1-tab-10.1177_1759720X211051471 for Rapid improvement in spinal pain in patients with axial spondyloarthritis treated with secukinumab: primary results from a randomized controlled phase-IIIb trial by Denis Poddubnyy, Effie Pournara, Agnieszka Zielińska, Asta Baranauskaite, Alejandro Muñoz Jiménez, Sanchayita Sadhu, Barbara Schulz, Michael Rissler, Chiara Perella and Helena Marzo-Ortega in Therapeutic Advances in Musculoskeletal Disease

sj-docx-2-tab-10.1177_1759720X211051471 – Supplemental material for Rapid improvement in spinal pain in patients with axial spondyloarthritis treated with secukinumab: primary results from a randomized controlled phase-IIIb trial Click here for additional data file. Supplemental material, sj-docx-2-tab-10.1177_1759720X211051471 for Rapid improvement in spinal pain in patients with axial spondyloarthritis treated with secukinumab: primary results from a randomized controlled phase-IIIb trial by Denis Poddubnyy, Effie Pournara, Agnieszka Zielińska, Asta Baranauskaite, Alejandro Muñoz Jiménez, Sanchayita Sadhu, Barbara Schulz, Michael Rissler, Chiara Perella and Helena Marzo-Ortega in Therapeutic Advances in Musculoskeletal Disease

sj-docx-3-tab-10.1177_1759720X211051471 – Supplemental material for Rapid improvement in spinal pain in patients with axial spondyloarthritis treated with secukinumab: primary results from a randomized controlled phase-IIIb trial Click here for additional data file. Supplemental material, sj-docx-3-tab-10.1177_1759720X211051471 for Rapid improvement in spinal pain in patients with axial spondyloarthritis treated with secukinumab: primary results from a randomized controlled phase-IIIb trial by Denis Poddubnyy, Effie Pournara, Agnieszka Zielińska, Asta Baranauskaite, Alejandro Muñoz Jiménez, Sanchayita Sadhu, Barbara Schulz, Michael Rissler, Chiara Perella and Helena Marzo-Ortega in Therapeutic Advances in Musculoskeletal Disease
