# Supplementary material for: Rapid improvement in spinal pain in patients with axial spondyloarthritis treated with secukinumab: primary results from a randomized controlled phase-IIIb trial
Source: Ther Adv Musculoskelet Dis. 2021 Oct 22;13:1759720X211051471. doi: 10.1177/1759720X211051471 (PMC8544765; doi:10.1177/1759720X211051471)
Supplement: sj-docx-2-tab-10.1177_1759720X211051471 – Supplemental material for Rapid improvement in spinal pain in patients with axial spondyloarthritis treated with secukinumab: primary results from a randomized controlled phase-IIIb trial [file sj-docx-2-tab-10.1177_1759720X211051471.docx]

**Supplementary figure S1. Study design**

**
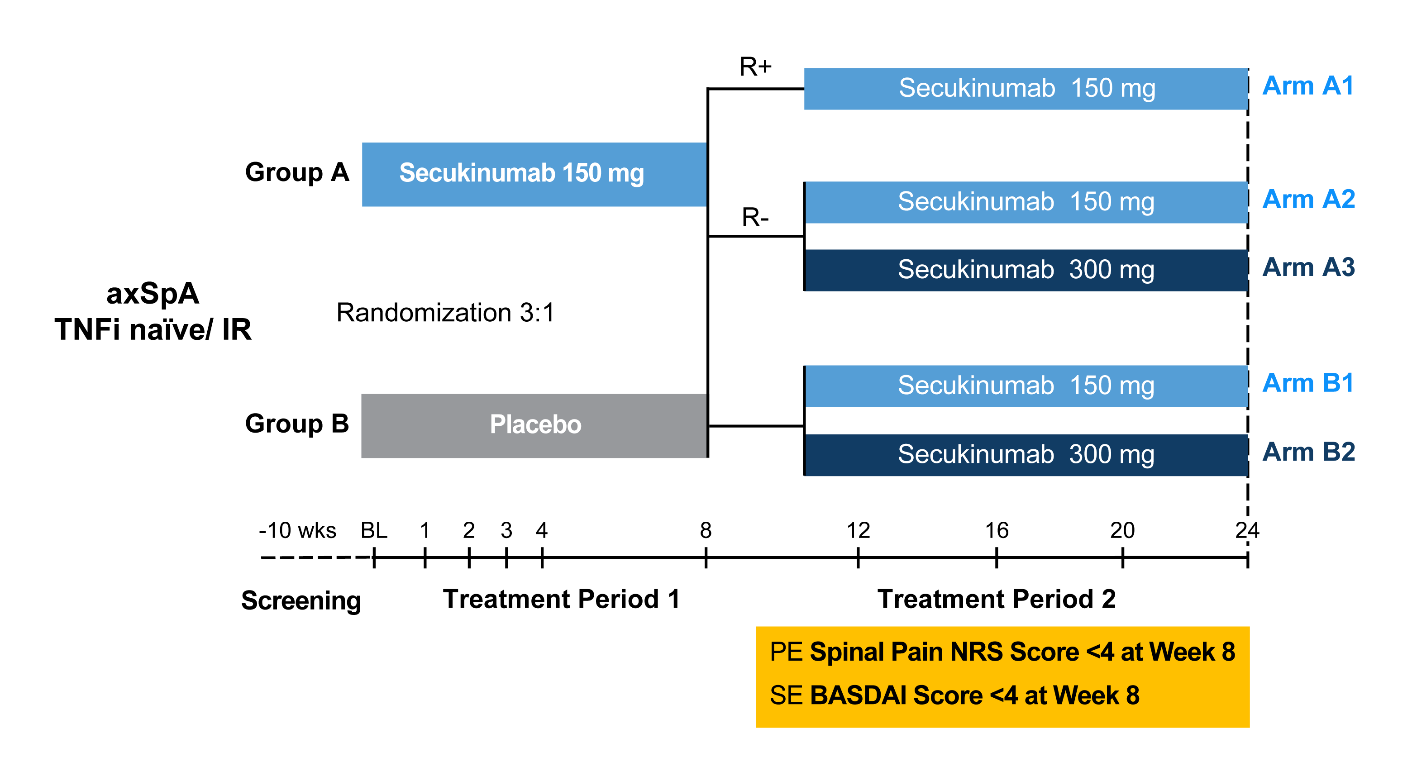
**

The study consists of two treatment periods:

- - **Treatment Period 1:** Placebo-controlled period from baseline to Week 8: secukinumab 150 mg or placebo
  - **Treatment Period 2:** Active treatment period from Week 8 to Week 24: secukinumab 150 mg or 300 mg

Patients were stratified at baseline based on whether they are naïve to TNFi or had an inadequate response (including intolerance) to TNFi.

axSpA, axial spondyloarthritis; BASDAI, Bath Ankylosing Spondylitis Disease Activity Index; BL, baseline, IR, inadequate responder; NRS, numerical rating scale; PE, primary end point; R+, responder, i.e., patient with spinal pain NRS score <4; R-, nonresponder, i.e., patient with spinal pain NRS score ≥4; SE, secondary end point, TNFi, tumor necrosis factor inhibitor; wks, weeks.

**Supplementary figure 2. Patient disposition**

**
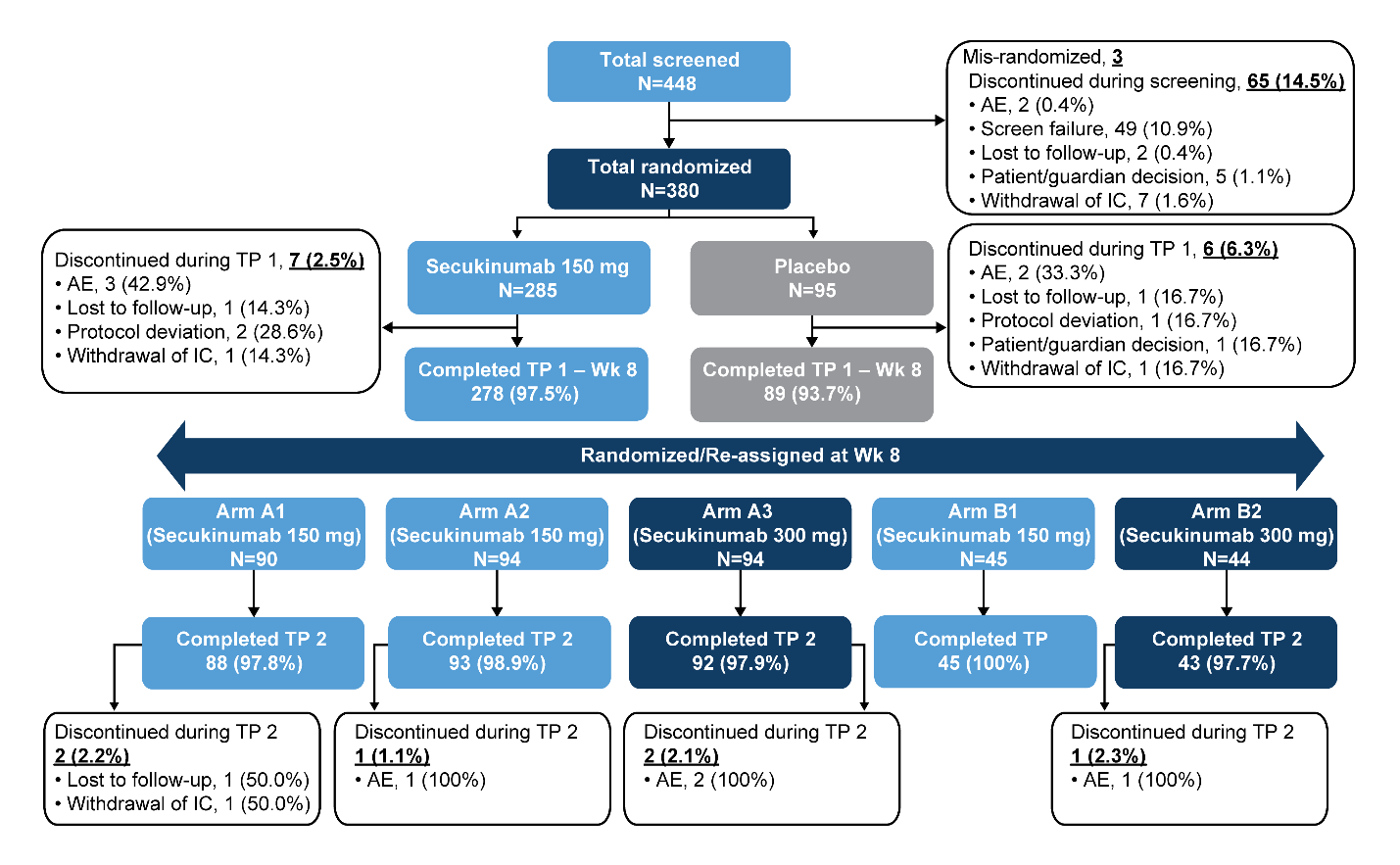
**

Discontinuation during screening was calculated based on the total screened patients (N=448)

| **TP 1** (up to Week 8) | **TP 2** (Week 8 - Week 24) |
| --- | --- |
| - SEC 150 mg | - *Arm A1*: SEC 150 mg responders (average spinal pain score <4) at Week 8 re-assigned to continue SEC 150 mg every 4 weeks |
|  | - *Arm A2*: SEC 150 mg nonresponders (average spinal pain score ≥4) re-randomized to continue SEC 150 mg every 4 weeks |
|  | - *Arm A3*: SEC 150 mg nonresponders (average spinal pain score ≥4) were up-titrated to SEC 300 mg every 4 weeks |
| - PBO | - *Arm B1*: Patients randomized to PBO at baseline were re-randomized to SEC 150 mg every 4 weeks |
|  | - *Arm B2*: Patients randomized to PBO at baseline were re-randomized to SEC 300 mg every 4 weeks |

AE, adverse event; IC, informed consent; N, total number of randomized patients; PBO, placebo; SEC, secukinumab; TP, treatment period; Wk, week.
