# Supplementary material for: Rapid improvement in spinal pain in patients with axial spondyloarthritis treated with secukinumab: primary results from a randomized controlled phase-IIIb trial
Source: Ther Adv Musculoskelet Dis. 2021 Oct 22;13:1759720X211051471. doi: 10.1177/1759720X211051471 (PMC8544765; doi:10.1177/1759720X211051471)
Supplement: sj-docx-3-tab-10.1177_1759720X211051471 – Supplemental material for Rapid improvement in spinal pain in patients with axial spondyloarthritis treated with secukinumab: primary results from a randomized controlled phase-IIIb trial [file sj-docx-3-tab-10.1177_1759720X211051471.docx]

**Supplementary table. Inclusion/Exclusion criteria**

| **Inclusion criteria** | **Exclusion criteria** |
| --- | --- |
| 1. Patients had to be able to understand and communicate with the investigator and comply with the requirements of the study and had to give a written, signed and dated informed consent before any study assessment was performed  2. Male or non-pregnant, non-nursing female patients of ≥18 years with diagnosis of axSpA according to ASAS axSpA criteria and with back pain for at least 3 months and age of onset <45 years  a. Sacroiliitis on imaging with ≥1 SpA feature  b. HLA-B27 positive with ≥2 SpA features  3. Active axSpA as assessed by total BASDAI ≥4 at baseline  4. Average spinal pain score >4 at baseline  5. Patients were to have been on ≥2 different NSAIDs at the highest recommended dose for ≥4 weeks in total prior to randomization with an inadequate response or failure to respond, or less if therapy had to be withdrawn due to intolerance, toxicity or contraindications  6. Patients who were regularly taking NSAIDs (including COX-1 or COX-2 inhibitors) as part of their axSpA therapy were required to be on a stable dose for at least 2 weeks before randomization  7. Patients who had previously been on a TNF-α inhibitor were allowed entry into study after an appropriate washout period prior to randomization:   - 4 weeks for Enbrel^®^ (etanercept) – with a terminal half-life of 102 ± 30 hours (s.c. route) - 8 weeks for Remicade^®^ (infliximab) – with a terminal half-life of 8.0–9.5 days (i.v. infusion) - 10 weeks for Humira^®^ (adalimumab) – with a terminal half-life of 10–20 days (average 2 weeks; s.c. route) - 10 weeks for Simponi^®^ (golimumab) – with a terminal half-life of 11–14 days - 10 weeks for Cimzia^®^ (certolizumab) – with a terminal half-life of 14 days | 1. Chest X-ray or MRI with evidence of ongoing infectious or malignant process, obtained within 3 months of screening and evaluated by a qualified physician (otherwise chest X-ray or MRI was required to be performed at screening) 2. Previous treatment with prohibited medications – etanercept, infliximab, adalimumab, golimumab, certolizumab, unstable dose of methotrexate or sulfasalazine, unstable or high dose of systemic corticosteroids, intramuscular/intra-articular/intravenous corticosteroids, conventional synthetic DMARDs other than MTX or sulfasalazine, leflunomide, targeted synthetic DMARDs, high strength opioids, analgesics other than NSAIDs or low strength opioids, tramadol and live vaccinations. Prohibited medications were to be washed out before randomization 3. Previous exposure to secukinumab or any other biologic drug directly targeting IL-17 or IL-23 4. Use of any investigational drug and/or devices within 4 weeks of randomization, or a period of 5 half-lives of the investigational drug, whichever was longer 5. History of hypersensitivity to the study drug or its excipients or to drugs of similar chemical classes 6. Patients previously treated with any biological immunomodulating agents, except those targeting TNF-α 7. Patients who had taken more than one TNFi agent 8. Previous treatment with any cell-depleting therapies including but not limited to anti-CD20 or investigational agents (e.g., CAMPATH^®^, anti-CD4, anti-CD5, anti-CD3, anti-CD19) 9. Pregnant or nursing (lactating) women, where pregnancy was defined as the state of a female after conception and until the termination of gestation, confirmed by a positive hCG laboratory test 10. Women of childbearing potential, defined as all women physiologically capable of becoming pregnant, unless they were using effective methods of contraception during entire study or longer if required by locally approved prescribing information (e.g., 20 weeks in European Union). In case of use of oral contraception, women should have been stable on the same pill for a minimum of 3 months before taking study treatment. Women were considered postmenopausal and not of childbearing potential if they had 12 months of natural (spontaneous) amenorrhoea with an appropriate clinical profile (e.g., age appropriate, history of vasomotor symptoms) or had surgical bilateral oophorectomy (with or without hysterectomy) or tubal ligation at least 6 weeks ago. In the case of oophorectomy alone, only when the reproductive status of the woman had been confirmed by follow-up hormone level assessment was she considered not of childbearing potential 11. Active ongoing inflammatory diseases other than axSpA that could confound the evaluation of the benefit of secukinumab therapy (such as inflammatory bowel disease and uveitis) 12. Other ongoing mechanical diseases affecting the spine (such as severe osteoarthritis of spine and diffuse idiopathic skeletal hyperostosis and fibromyalgia) that could confound evaluation of the benefit of secukinumab therapy 13. Underlying metabolic, haematological, renal, hepatic, pulmonary, neurological, endocrine, cardiac, infectious or gastrointestinal conditions, which, in the opinion of the investigator, immunocompromised the patient and/or placed the patient at unacceptable risk for participation in an immunomodulatory therapy 14. Significant medical problems or diseases, including but not limited to the following: uncontrolled hypertension (≥160/95 mmHg), congestive heart failure (New York Heart Association status of class III or IV), uncontrolled diabetes or very poor functional status (unable to perform self-care) 15. History of clinically significant liver disease or liver injury as indicated by abnormal liver function tests such as aspartate aminotransferase, alanine aminotransferase, alkaline phosphatase or serum bilirubin. The investigator was to be guided by the following criteria:  - Any single parameter was not to exceed 2× ULN. A single parameter elevated up to and including 2× ULN was to be re-checked once more as soon as possible, and in all cases, at least prior to enrolment/randomization, to rule out lab error. - If the total bilirubin concentration was increased above 2× ULN, total bilirubin was to be differentiated into the direct and indirect reacting bilirubin.  1. History of renal trauma, glomerulonephritis or patients with one kidney only, or a serum creatinine level exceeding 1.5 mg/dL (132.6 μmol/L) 2. Screening total white blood cell count <3000/μL, or platelets <100,000/μL or neutrophils <1500/μL or haemoglobin <8.5 g/dL (85 g/L) 3. Active systemic infections during the last 2 weeks prior to randomization (exception: common cold) 4. History of ongoing, chronic or recurrent infectious disease or evidence of tuberculosis infection as defined by a positive QuantiFERON TB-Gold test. Patients with a positive test could participate in the study if further work-up (according to local practice/guidelines) established conclusively that the patient had no evidence of active tuberculosis. If presence of latent tuberculosis was established, then treatment according to local country guidelines had to be initiated according to local guidelines 5. Known infection with HIV, hepatitis B or hepatitis C at screening or randomization 6. History of lymphoproliferative disease or any known malignancy or history of malignancy of any organ system within the past 5 years (except for basal cell carcinoma or actinic keratoses that had been treated with no evidence of recurrence in the past 3 months, carcinoma *in situ* of the cervix or noninvasive malignant colon polyps that had been removed) 7. Any current severe progressive or uncontrolled disease that, in the judgment of the clinical investigator, rendered the patient unsuitable for the trial 8. Any medical or psychiatric condition that, in the investigator’s opinion, would have precluded the participant from adhering to the protocol or completing the study per protocol 9. History or evidence of ongoing alcohol or drug abuse, within the last 6 months before randomization 10. Administration of live vaccines 6 weeks prior to randomization or plans for administration of live vaccines during the study period; live vaccines should not have been given until 12 weeks after last study treatment administration |

AS, ankylosing spondylitis; ASAS, Assessment in SpondyloArthritis international Society; axSpA, axial spondyloarthritis; BASDAI, Bath Ankylosing Spondylitis Disease Activity Index; COX, cyclooxygenase; DMARDs, disease-modifying antirheumatic drugs ; hCG, human chorionic gonadotropin; HIV, human immunodeficiency virus; HLA, human leukocyte antigen; IL, interleukin; MRI, magnetic resonance imaging; MTX, methotrexate; NSAIDs, nonsteroidal anti-inflammatory drugs; TNFi, tumor necrosis factor inhibitor; ULN, upper limit of normal**.**
